# Progressive Resistance Exercise with Eccentric Loading for the Management of Knee Osteoarthritis

**DOI:** 10.3389/fmed.2015.00045

**Published:** 2015-07-09

**Authors:** Haniel J. Hernandez, Valerie McIntosh, Azadeh Leland, Michael O. Harris-Love

**Affiliations:** ^1^Human Performance Research Unit, Muscle Morphology, Mechanics and Performance Laboratory, Clinical Research Center, Veterans Affairs Medical Center, Washington, DC, USA; ^2^Physical Medicine and Rehabilitation Service, Veterans Affairs Medical Center, Washington, DC, USA; ^3^Department of Exercise and Nutritional Sciences, Milken Institute School of Public Health, The George Washington University, Washington, DC, USA

**Keywords:** knee arthritis, strengthening, eccentric exercise, veterans, ultrasound

## Abstract

**Introduction:**

The patient was a 58-year-old African-American male with radiographic evidence of bilateral knee osteoarthritis (OA). He participated in a standardized 12-week eccentric strengthening program within a Veterans Affairs (VA) medical center.

**Background:**

The use of an eccentric training paradigm may prove to be beneficial for older adults with knee OA since eccentric muscle actions are involved in the energy absorption at the knee joint during gait and controlled movement during stair descent. Furthermore, in comparison to standard muscle actions, eccentric muscle actions result in higher torque generation and a lower rate of oxygen consumption at a given level of perceived exertion. Therefore, this mode of progressive resistance exercise may be ideal for older adults.

**Discussion:**

The patient completed an eccentric strengthening regimen for the knee flexors and extensors twice per week without an exacerbation of knee pain. Muscle morphology measures of the rectus femoris were measured using diagnostic ultrasound. Isokinetic measures of muscle peak torque were obtained at 60°/s and 180°/s. Functional performance was assessed using a physical performance battery and stair-step performance was assessed from the linear displacement of the center of gravity trajectories obtained with a force plate. Visual analog scale pain ratings and self-reported global disease status were also documented. Post-exercise assessments revealed improvements in sonographic muscle size and tissue composition estimates, peak knee extensor torque (ranging from 60 to 253%), functional performance, and global disease status.

**Concluding remarks:**

The patient exhibited improvements in muscle morphology, muscle strength, functional performance, pain, and global disease status after 12 weeks of an eccentric strengthening regimen. The intervention and outcomes featured in this case were feasible to implement within a VA medical center and merit further investigation.

## Introduction

Given the increased average life expectancy due to advancements in technology and medical care, chronic conditions such as osteoarthritis (OA) may have an impact on the aging population regarding their performance of daily tasks and health-related quality of life (HRQL). OA is the most prevalent form of OA (1), and the knee is one of the largest joints most frequently affected by it (2). The current view of OA is that it extends beyond subchondral bone changes and cartilage degeneration, and also involves extra-articular tissue such as muscles, ligaments, and afferent sensory nerves (3). Knee OA may result in muscular weakness, particularly in the quadriceps (4). Additionally, underutilization of the musculature surrounding the knee due to pain from walking or other physical activity may contribute to knee weakness through deconditioning and disuse atrophy. Consequently, knee OA may lead to a decrease in activity levels, aberrant gait mechanics, and increased fall risk (5). Recent work suggests that high levels of joint loading associated with intense sports activities such as elite-level weightlifting and amateur-level soccer and football are associated with higher relative risk of developing knee OA (6), whereas other investigators have found that formal exercise programs do not increase disease risk or exacerbate knee OA symptoms (7, 8).

Physical therapy is the conservative approach for addressing the sequalae associated with knee OA, and progressive resistive exercise along with manual therapy techniques to improve joint arthrokinematics are often employed within the plan of care. While investigators have noted that physical therapy features effective intervention approaches for knee OA, it is important to note that these interventions do not only entail exercise (9, 10). Nevertheless, therapeutic exercise has been shown to promote pain relief and improve global disease status ratings for people with OA (11, 12), and strength training is associated with knee OA pain relief with or without other modes of exercise such as aerobic training (13). However, there is no clear evidence concerning the mode of strengthening exercise and the optimal parameters for exercise intensity.

The benefits of strength training for knee OA has been studied extensively over the past two decades (8, 10, 11, 14–16). Preliminary evidence supports the use of isokinetic exercise as a mode of strength training in the management of knee OA (17, 18). Previous isokinetic protocols have featured the use of sequential or reciprocal concentric/eccentric muscle actions (19–21). Eccentric muscle actions can be described as a muscle lengthening while it is under tension and producing force. The eccentric muscle action of the quadriceps plays a role in everyday activities such as descending stairs and walking. Early findings suggest that combined concentric/eccentric isokinetic strengthening may result in greater functional improvement in people with knee OA in comparison to a concentric-only isokinetic exercise (21). However, further investigation of this approach to therapeutic exercise will be required to understand if eccentric exercise has meaningful advantages over other modes of progressive resistance exercise (PRE) in the treatment of knee OA. Various modes of PRE featuring eccentric muscle actions have been used as a form of therapeutic exercise for conditions ranging from Achilles tendinopathy to cardiopulmonary disease (22). Nevertheless, the use of eccentric-only isokinetic exercise as a mode of PRE for people with knee OA remains largely unexplored. This case report details the response of a 58-year-old veteran with bilateral knee OA to a 12-week standardized regimen of isokinetic eccentric PRE. The objectives of this work included conducting a detailed account of exercise tolerance and response to eccentric PRE in a patient with significant knee OA, and exploring the utility of selected outcomes to measure the presumed adaptations to the exercise stimulus.

### Case description

#### Patient History

This study was approved by the Institutional Review Board of the Research & Development Service at the Washington DC Veterans Affairs (VA) Medical Center and informed consent was obtained from the patient. The patient featured in this case report was a 58-year-old male veteran with a history of chronic left knee pain over the course of the past 4–5 years. His primary complaints included knee joint stiffness, soreness, with intermittent swelling. The patient stated that he engaged in limited physical activity, with sporadic bouts of formal exercise approximately “1–2 times every few weeks.” However, he worked in an office, which required manual labor that would vary from 20 to 40% of his total hours. The patient reported that his biggest challenge was negotiating stairs, with the worst exacerbation of pain occurring while descending stairs. He also had difficulty with standing up after sitting down for periods longer than an hour, and pain with walking after 5–10 min. The patient participated in physical therapy for the knee pain approximately 2 years prior, and reported that the rehabilitation regimen was very effective in the management of his knee OA. However, following his discharge from physical therapy, he noted that his left knee pain had gradually returned after 4–5 months. The patient stated that he was not involved in any formal exercise program or receiving any physical therapy care at the time of his evaluation.

#### Initial Examination and Evaluation

Left knee pain was rated as 6 out of 10 using a standard numeric pain scale at the time of the initial examination. The patient had no complaints of any problems with the right knee joint. An antalgic gait favoring the left knee was noticeable upon visual gait assessment and observation. Left knee flexion active range of motion (AROM) was found to be 120° and extension AROM to be 0°. Physical appearance of the knees was characterized by normal alignment with no notable excessive valgus or varus positioning. No swelling or edema was present upon visual inspection. There was notable crepitus with passive ROM into flexion and extension that was noticeable by both the patient and clinician. No hypersensitivity or tenderness was elicited with palpation at the joint line. There was some reported discomfort with tibial internal and external rotation at 90° of knee flexion. Special tests were performed to rule out any meniscal or ligamentous sources of pain or dysfunction. Apley’s and McMurray’s tests were both found to be negative for meniscal injury. Valgus and varus stress tests were negative, indicating no issues with the medial collateral and lateral collateral ligaments, respectively. Clarke’s test for chondromalacia patella was positive. The only notable manual muscle test (23) differences were with knee extension assessed in unsupported sitting where the left lower extremity was scored as 3+/5 and the right lower extremity was scored as 5/5. Deep tendon reflexes at patellar tendon were normal at +2 bilaterally along with sensation being within normal limits. The rheumatologist reviewed the radiographs and determined that the patient had bilateral knee OA. It was determined that the most involved joint, the left knee, had grade 3 OA based on the Kellgren–Lawrence classification. A grade 3 classification is defined as moderate osteophyte formation, definite joint space narrowing, and the presence of some sclerosis and joint deformity (24). The patient denied having rheumatoid arthritis or previous knee surgeries. Apart from his formal diagnosis of OA, bilateral pes planus was the only other musculoskeletal problem detected during the examination. He had no notable cardiovascular disease, pulmonary dysfunction, or diabetes based on the chart review and the provided medical history.

## Background

### Intervention

**Figure 1:**
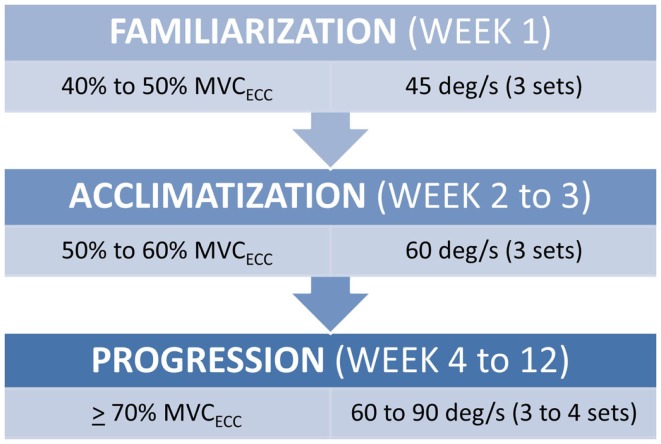
**Submaximal eccentric progressive resistance exercise phases**. The training regimen features structured phases of submaximal eccentric exercise designed to safely progress the strength training stimulus in people with musculoskeletal impairments. (MVC_ECC_, estimated eccentric maximum voluntary contraction, i.e., concentric MVC at 60°/s × 1.35).

All strength assessment and eccentric PRE was performed using an isokinetic dynamometer (Biodex System 4, Biodex Medical Systems, Shirley, NY, USA). The eccentric PRE regimen was 12 weeks in duration with training scheduled twice per week with at least 1 day of rest between sessions. The progression of the eccentric PRE workload was adapted from our previously described method of isokinetic eccentric-only strengthening exercise (25). Briefly, the isokinetic eccentric PRE periodization scheme (Figure 1) is based on three training phases that facilitate the use of eccentric exercise in a manner suitable for patient populations. The first stage of training is the *familiarization phase*. This phase of training is characterized by low exercise intensity (i.e., the target exercise torque expressed as a percentage of the estimated maximum eccentric peak torque) and training volume. The familiarization phase allows time for motor learning regarding the unique muscle recruitment patterns associated with isokinetic eccentric exercise; it fosters consistency in exercise performance, and it provides an opportunity for the physical therapist to correct flaws in exercise form. The second stage of training is the *acclimatization phase*. This phase is noted by a progressive, but moderate, increase in exercise intensity with target exercise torque levels sufficient to induce the “repeated bout effect” – where previous muscle actions are protective against excessive muscle damage and delayed onset muscle soreness (DOMS) from subsequent muscle actions at equal or higher exercise intensity (26, 27). Finally, the third stage of training is the *progression phase*, which is marked by incremental exercise intensity at levels presumed to optimally induce training adaptations while maintaining patient safety.

The progression phase features a training algorithm that allows for the modification of the training workload each session. This modification may result in exercise intensity remaining stable between sessions, or increasing by 5% if all torque targets are met (across all PRE sets), or decreasing by 10% if the torque targets are not met due to excessive fatigue (i.e., attainment of ≤70% of the target exercise torque during any PRE set). All exercise sets included 10 repetitions with the exercise volume progressing from three to four sets by Week 6, and the progressive phase movement speed transitioning from 60°/s to 90°/s by Week 10. The completion of each set was proceeded by 1 min of recovery time. The patient was able to monitor his ability to meet the torque targets with each repetition by viewing the torque-time curves on the Biodex computer monitor during the exercise bouts. Given our interest integrating eccentric PRE into the plan of care for patient populations with significant impairments, we have elected to refrain from maximally testing eccentric strength in people who are naïve to the training stimulus and/or managing active musculoskeletal or neuromuscular disease. Consequently, the isokinetic concentric maximum voluntary contraction (MVC) at 60°/s was used to derive the estimated eccentric peak torque. This estimate was obtained using the following equation (where, MVC expressed as torque, Ecc = eccentric, and Con60 = concentric at 60°/s):

$${\mathrm{MVC}}_{\mathrm{Ecc}}={\mathrm{MVC}}_{\mathrm{Con60}}\times 1.35.$$

This equation includes a minor adjustment of the previously used co-factor of 1.4 since its initial use did not include patients with joint pain (25).

### Outcome measures

#### Measures of Impairment

Outcome measures featured in this report were utilized at Week 1 and 12 of the eccentric PRE regimen, and included multiple domains of disablement. Pain was documented using a written, self-reported, visual analog scale (VAS) based on a numeric scale of measurement (VAS; 0–10 scoring range) (28) with separate ratings obtained for knee joint pain and lower extremity muscle pain. Pain ratings were obtained prior to each exercise bout, and instructions were provided to the patient to indicate if pain levels increased during or immediately after exercise. Strength of the knee flexors was assessed via isokinetic dynamometry at 60°/s and 180°/s using methods adapted from previously published protocols (25, 29) with patient positioning and stabilization per the Biodex Operations Manual. Following a familiarization session, the MVC was derived from the mean value of the highest three peak torque values from a five repetition test. Previous investigators have found this form of strength assessment to be reliable (29) (intraclass correlation coefficients, ICCs, exceeding 0.92), and standard error of measurement (SEM) is approximately 8% in older adults (30). Diagnostic musculoskeletal ultrasound (SonoSite, Inc., Bothell, Washington, DC, USA) was used to characterize muscle morphology and morphometry at the mid-point of the rectus femoris before and after the eccentric PRE regimen. Longitudinal ultrasound image capture (6 cm depth) was used to obtain muscle thickness and echointensity values. Each measure was completed three times and the mean values were used for pre and post-exercise comparisons. Ultrasound measures of muscle thickness have been used to estimate post-exercise muscle hypertrophy (31) and measures of echointensity via grayscale analysis have been associated with muscle quality in older adults (32). Acceptable intra-rater reliability (33, 34) for diagnostic ultrasound assessment has been found for tests involving the thickness and cross-sectional area of the rectus femoris (ICC_3,_ *_k_* = 0.72–0.99, *p* < 0.05). Also, the physical therapists involved in this case have demonstrated a CV of 1.6–2.9% for material thickness measures across multiple raters using a calibration phantom, and an ICC_2,_ *_k_* of 0.99 (95% C.I. = 0.96–0.99, *p* < 0.001; SEM = 1.3–1.6) for the assessment of tissue echointensity at the rectus femoris in a cohort of older adults (35).

#### Measures of Functional Performance

The patient’s ability to ascend and descend a step was objectively assessed using the Neurocom^®^ Step Up and Over test (Neurocom International, Clackamas, OR, USA). This test involves starting in stance using a standardized location on a force plate, ascending an 8-inch high step with the ipsilateral limb, transitioning from step ascent to step descent while advancing the trailing contralateral limb, and then ending the test when both feet have returned to the force plate. This test documents elements of performance such as movement time and center of gravity (COG) displacement based on three consecutive trials for each leg. Mean values for movement time were obtained from the Neurocom^®^ EquiTest software, and the mean linear COG displacement trajectories were measured using ImageJ (NIH, Bethesda, MD, USA; Ver. 1.48) to determine the maximum distance traversed during the Step Up and Over test. While the Neurocom^®^ Step Up and Over test has been featured in previous validity studies (36–38), the clinimetric properties of the test have not been subject to extensive study. However, the Step Up and Over test was found to have fair to excellent reliability (ICC_3,_ *_k_* = 0.59–0.92) in a sample of physically active young women (39). The Step Up and Over test was chosen as an outcome measure in order to obtain objective information related to a common lower extremity task encountered in home and community environments. Moreover, the descent phase of the Step Up and Over test is a movement dependent on eccentric muscle control at the knee joint. Our premise was that the COG displacement would be reduced when ascending and descending the step using the more involved limb secondary to joint pain and/or impaired muscle strength. General physical performance was assessed using the modified Physical Performance Test (PPT-7). The PPT-7 is an observed, performance-based battery that has been validated for use with older adults (40). The test battery is comprised of the following items: writing a sentence, simulated eating, turning 360°, putting on and removing a jacket, lifting a book and putting it on a shelf, picking up a penny from the floor, and a 50-foot walk test. Both a Likert scale rating and continuous measures using a timer are used to score the test battery. Therefore, habitual walking speed was also obtained from the patient’s performance of the walk test (Item #7) during the completion of the PPT-7. The cut-off score for moderate frailty is a total score of less than 19.4 (scoring range, 0–28) and the test inter-rater reliability is suitable for clinical practice (40–42).

#### Measures of Global Disease Status

The Knee Injury and Osteoarthritis Outcome Score (KOOS) was selected as an outcome measure for OA disease status and HRQL. The construct validity (43) of the KOOS is ideal for the VA medical center setting since it is designed to address knee symptoms secondary to traumatic orthopedic injuries, which often result in the medical separation of soldiers from active duty, and age-related degenerative arthritic conditions, which frequently affect older veterans (44). The KOOS consists of five subscales; Pain, Symptoms, Activities of Daily Living (ADL), Sport and Recreation Function (Sport/Rec), and knee-related Quality of Life (QOL). The previous week is the time period considered when completing the self-administered questionnaire. Standardized answer options are given (based on a 5-point Likert scale) and each question is assigned a score from 0 to 4. A normalized score is calculated for each subscale (a score of 100 indicates “no symptoms”, and a score of 0 indicates “extreme symptoms”). The KOOS has acceptable reliability (45) with intraclass correlation coefficients that range from 0.75 to 0.95 for all subscales, with the exception of the Sport/Rec subscale (0.61–0.89). A minimal perceptible clinical improvement (MPCI) of 8–10 points has been proposed (43), and the minimal detectable changes (MDC) (46) for the subscales may range from 13.4 to 21.1.

## Discussion

### Results

**Figure 2:**
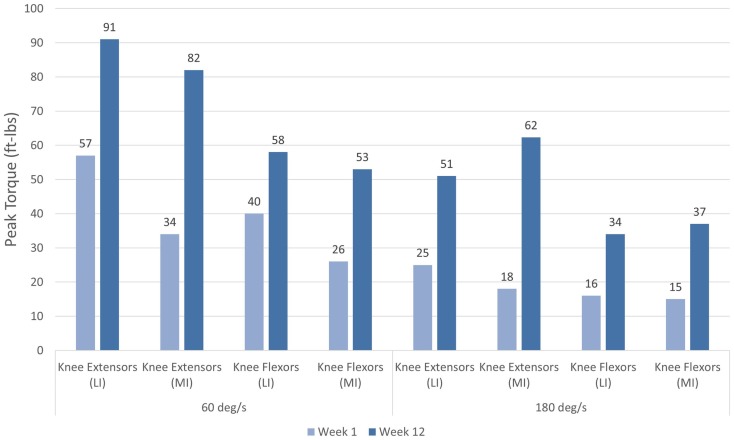
**Pre and post-intervention concentric knee extensor and flexor peak torque values**. Increases in concentric knee extensor and flexor performance were noted following the 12-week eccentric progressive resistance exercise regimen. The adaptations to the eccentric strength training stimulus were transferable to the concentric muscle actions across both muscle groups and movement speeds. The greatest magnitude of strength change was observed in the more involved limb (left side).

**Figure 3:**
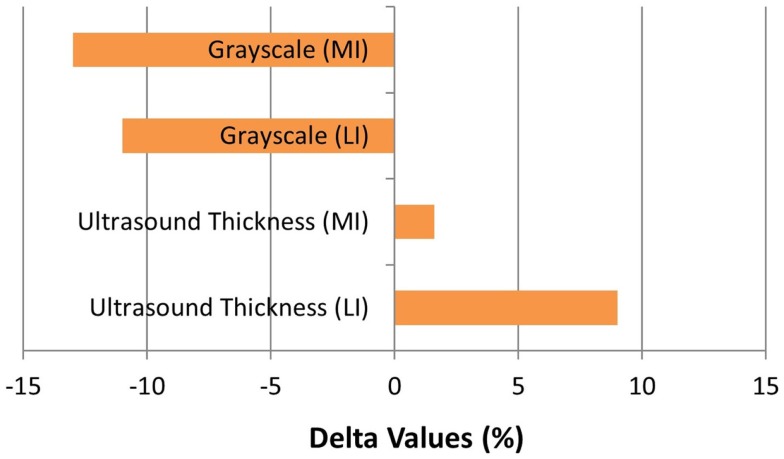
**Muscle morphology adaptations following the 12-week intervention period**. The grayscale delta percentage values shown at the top of the graph depict the decreased echogenicity estimates from the ultrasound images of the rectus femoris, which is suggestive of improved tissue composition following the eccentric exercise regimen. The ultrasound muscle thickness delta percentage values depicted at the bottom of the graph suggest that muscle hypertrophy occurred, primarily in the less involved limb (right side), following the 12-week intervention period (MI, more involved limb; LI, less involved limb).

**Figure 4:**
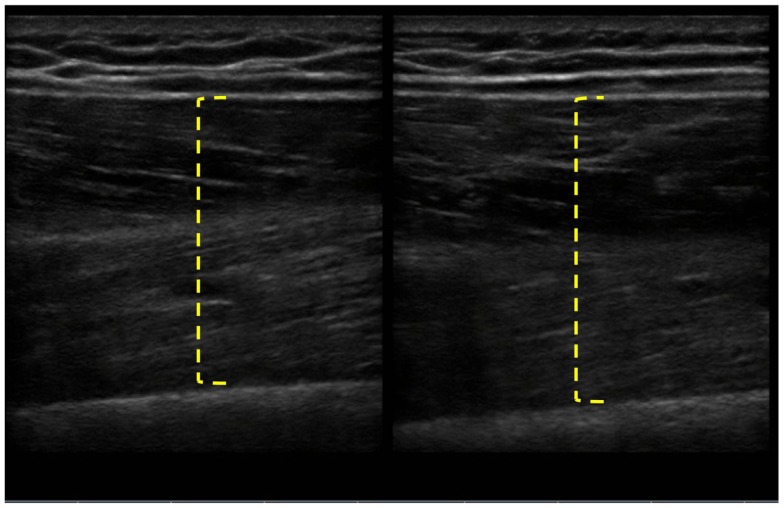
**Pre and post-intervention ultrasound images of the rectus femoris following the 12-week intervention period**. The longitudinal B-mode ultrasound images of the less involved limb (right side) were obtained with a 13–6 MHz linear array transducer and using the averaged digital caliper values from three measurements. The rectus femoris of the less involved limb had an increase in muscle thickness from 3.97 to 4.32 cm, whereas the more involved limb experienced a change in muscle thickness of <0.1 cm.

The patient completed all of the training sessions without any exacerbation of pain during or after the exercise bouts. The exercise sessions lasted approximately 20–30 min. The exercise target torques (i.e., the torque goal for each 10-repetition set) for the knee extensors at the start of the progressive phase (Week 4) were 54 ft-lbs on the less involved limb (right side) and 32 ft-lbs on the more involved limb (left side). At the end of the progressive phase (Week 12) the patient advanced his exercise target torques to 112 ft-lbs on the less involved limb and 76 ft-lbs on the more involved limb. These final torque targets during the eccentric PRE for the less involved and more involved limbs were 107 and 138%, respectively, higher than the pre-exercise isokinetic concentric MVC for the knee extensors. All of the pre and post-exercise isokinetic MVC values are summarized in Figure 2. The less involved limb consistently exhibited a greater magnitude of increased strength across both testing conditions and muscle groups. Strength improvements in the less involved limb ranged from 44 to 115%. By contrast, more involved limb exhibited strength improvements that ranged from 104 to 253%. The highest absolute MVC values were generated by the less involved limb in both testing conditions during the pre-exercise assessment. However, post-exercise MVC values of the more involved limb were higher than those in the less involved limb in the 180°/s testing condition. The pre and post-exercise changes in the patient’s muscle thickness and echointensity at the rectus femoris are presented in Figure 3. Ultrasound examinations required approximately 10–15 min to complete. A uniform post-exercise decrease in echointensity was noted for both lower limbs, whereas the increase in muscle thickness was primarily in the less involved limb. Thickness for the rectus femoris in the less involved limb increased from 3.97 to 4.32 cm following 12 weeks of eccentric PRE (Figure 4). However, muscle thickness in the more involved limb was less pronounced, increasing from 3.65 to 3.71 cm over the same time period.

**Figure 5:**
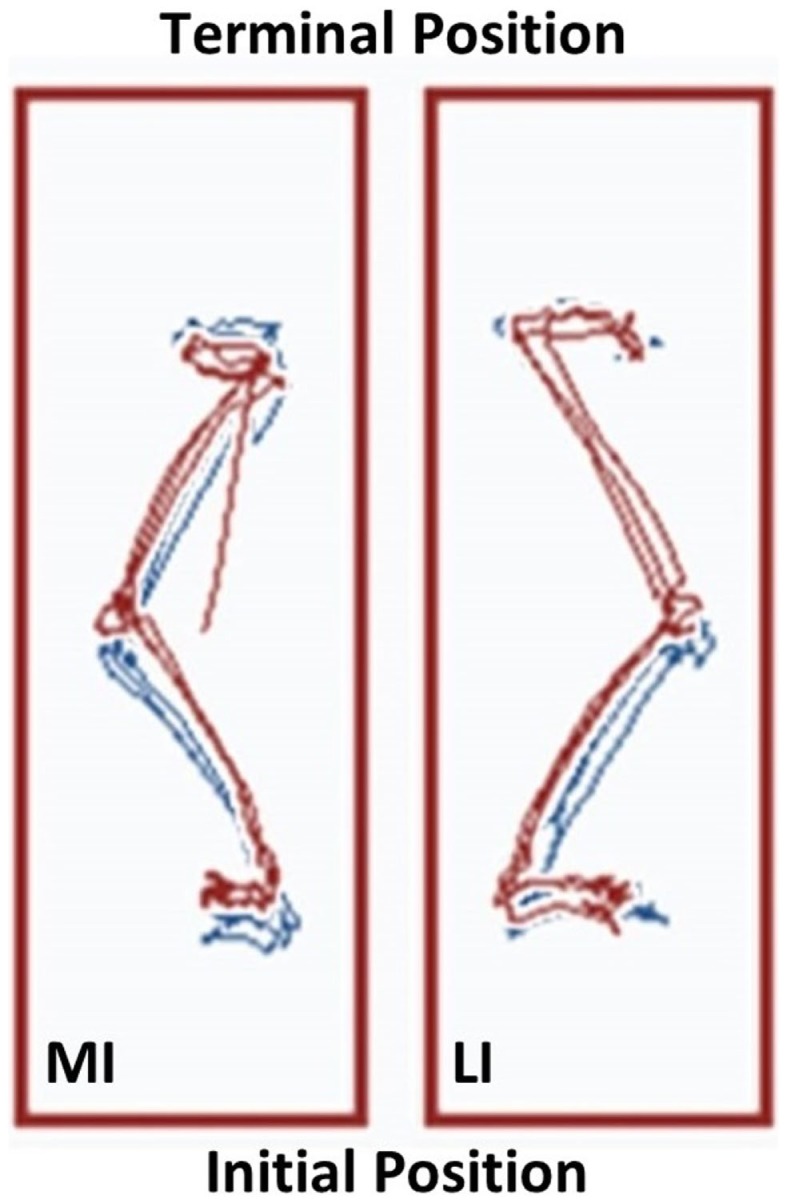
**The Neurocom^®^ Step Up and Over test performance following the 12-week intervention period**. The center of gravity (COG) displacement trajectories derived from the Step Up and Over test are displayed for the more involved limb (left side) and the less involved limb (right side). The Week 12 trajectories are in blue and overlaid with the Week 1 trajectories in red. The trajectories on the right have a maximum linear COG displacement that differs by <2% for the less involved limb. In contrast, the trajectories on the left show that the linear COG displacement of the more involved limb increased by 13.7% after the intervention period. This observation is consistent with a longer stepping length for the more involved limb, which resulted in improved symmetry between sides (MI, more involved limb; LI, less involved limb).

Performance of the Step Up and Over test also reflected potential differences between the lower extremities. The linear displacement of the COG trajectory on the less involved limb was 1.11 m at the start of the exercise regimen and 1.14 m at Week 12. In comparison, the more involved limb exhibited linear displacement COG trajectory values that increased from 1.02 to 1.16 m over the 12-week period. As a result, the asymmetry in the Step Up and Over test performance decreased from 8.8 to 1.7%. The task performance time was similar between the lower extremities pre and post-exercise with the less and more involved limbs attaining 1.83 ± 0.05 s and 1.86 ± 0.26 s mean values, respectively, at Week 12. However, the longest duration single trial task performance time was observed during Week 12 in the more involved limb (2.10 s for the left leg versus 1.87 s for the right leg). The COG trajectories are shown in Figure 5.

The patient’s general physical performance was observed using the PPT-7 test battery. He demonstrated modest improvements as his score increased from 19 during Week 1 to 21 during Week 12. Gait speed was also examined independently from the other PPT-7 items to further characterize the patient’s physical status. Habitual walking speed is a widely recognized as a key functional measure and an indicator of health status in older adults (47, 48). The patient’s gait performance improved by 16% as his speed increased from 0.88 to 1.02 m/s after the eccentric PRE regimen.

All of the KOOS subscale post-exercise values were improved in comparison to the baseline values. The pre and post-exercise scores are presented in Table 1. Interpretation of the changes in the KOOS subscale scores was aided by use of the MPCI and the MDC values (see Table 1). The KOOS subscales with the largest magnitude of change following the eccentric PRE regimen were the Sport/Rec and QOL subscales. These measures were also the only subscales with change scores that exceeded both the MPCI and MDC criteria. While the Symptom subscale improved from Week 1 to 12, the observed seven-point change did not meet either the MPCI or MDC criteria. In addition, the post-exercise Pain subscale improved by eight points and met the MPCI criteria while the VAS pain rating remained stable during the entire 12-week period.

**Table 1:** **Pre and post intervention Knee Injury and Osteoarthritis Outcome Score (KOOS) subscale scores**.

KOOS subscales	Week 1	Week 12	MDC/MPCI
Pain	42	50	−/+
Symptoms	57	64	−/−
ADL	40	50	−/+
Sport/Recreation	5	40	+/+
QOL	0	38	+/+

The patient exhibited post-exercise increases in all of the KOOS subscales. However, the improvements met the criteria of the MDC and PMCI only in the Sport/Recreation and QOL subscales.

ADL, activities of daily living; QOL, quality of life; MDC, minimal detectable change values: Pain, 13.4; Symptoms, 15.5; ADL, 15.4; Sport/Rec, 19.6; QOL 21.1; MPCI, minimal perceptible clinical improvement: ≥8.

### Clinical implications

The progression of knee OA is characterized by a group of symptoms that may include joint pain, lower extremity weakness, gait disturbances, and a general loss of endurance secondary to the activity limiting pain (49–51). While combined concentric/eccentric strengthening programs have been used to address these OA symptoms (19–21), the use of eccentric-only strength training approaches for this patient population requires further investigation. In this case report, a 58-year-old veteran with bilateral knee OA exhibited improvements across disablement domains following a 12-week standardized regimen of isokinetic eccentric PRE.

**Figure 6:**
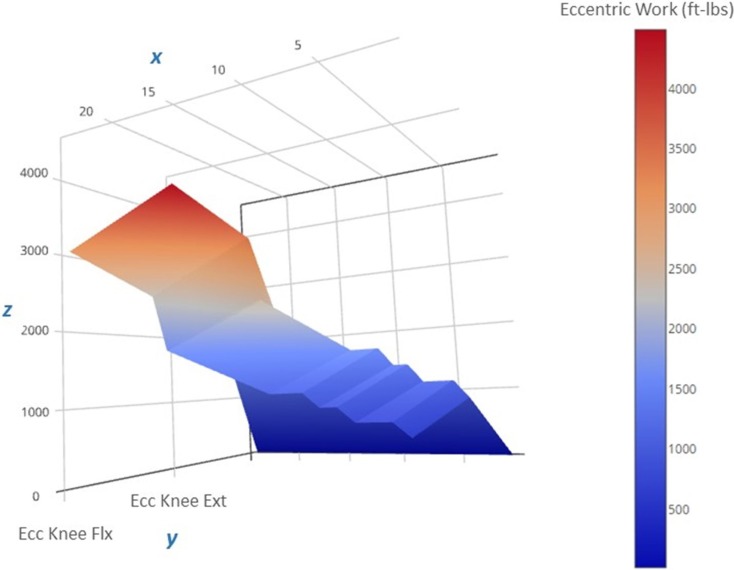
**Progression of the estimated eccentric work volume produced during the 12-week intervention period**. The 3-D surface plot depicts the estimated unilateral eccentric (Ecc) work produced by the less involved limb (right side) knee extensors over the course of the exercise regimen. The work produced is a function of the isokinetic torque produced during knee excursion and the exercise volume (in repetitions and sets). The *x*-axis features exercise sessions (two per week, for a total of 24 sessions), the *y*-axis includes the agonist muscle groups (knee flexors and extensors), and the *z*-axis includes the estimated work yield per exercise session (ft-lbs). Note that the lower work volume at the start of the regimen (the dark blue region on the right side of the plot) corresponds to the lower torque targets that characterize the “Familiarization” and “Acclimatization” phases of the eccentric exercise regimen, and may have afforded a protective adaptation against excessive muscle damage during the subsequent exercise bouts marked by higher work volumes and torque levels during the final 4 weeks of the “Progression” phase (the red region on the left side of the plot).

An important observation from this case study is the ability of a patient with significant knee pain (VAS = 6 out of 10 at the start of the program) to engage in an eccentric PRE regimen without any exacerbation of his symptoms. Moreover, the patient did not report any DOMS despite the general concerns about excessive muscle damage secondary to eccentric exercise for specific musculoskeletal conditions (22) and the known initial adverse response to supramaximal eccentric exercise (52). In this case report, the initial submaximal eccentric training intensity featuring a phased approach to workload (Figure 6) may have sufficiently induced the “repeated bout effect” thus affording some protection from myofibrillar disruption and excessive local inflammation (25, 27). The patient’s eccentric PRE torque targets gradually exceeded his concentric MVC by Week 6 of the program. This progressive overload scheme allowed the patient to attain workload levels that far exceed what would be achievable with an isokinetic concentric approach or a standard isoinertial program over a similar period of time. The qualities of eccentric muscle actions such as greater metabolic efficiency (53, 54) and higher torque production (55) in comparison to concentric actions were adapted and implemented for rehabilitation purposes in the treatment of the patient.

The patient appeared to have a meaningful response to the eccentric PRE stimulus given the magnitude of change reflected in the post-exercise MVC values. However, it is important to note that the age of the patient featured in this report was 58 years old, which is younger than the median age of U.S. veterans (62 years of age) and outside the NIH National Institute of Aging’s category of the “young-old” (65–80 years of age) (56, 57). Therefore, a less robust response to the exercise regimen could occur in adults over the age of 65 with knee OA and in those with significant comorbidities.

Since eccentric PRE was used as the sole strength training regimen for the patient during the intervention period, the strength assessment measures at Week 12 may represent the transference of eccentric muscle action adaptations to concentric muscle actions. While the specificity of training applies to all forms of muscle action, the transference of the physiologic adaptations to eccentric exercise to isometric or concentric muscle actions have been previously noted (25, 58). These physiologic adaptations to an eccentric training stimulus were consistent with our observation of the patient’s post-exercise changes in morphology and morphometry of the rectus femoris. Increases in rectus femoris muscle thickness, which are indicative of muscle hypertrophy, and possible improvements in muscle quality based on the lower ­echointensity values were noted in the patient’s Week 12 measures and are consistent with previously reported findings in healthy younger adults (59). There was a disparate response in adaptive muscle hypertrophy in the patient as the rectus femoris size increased by 9% in the less involved limb and 1.6% in the more involved limb. This difference between the limbs was also reflected in the MVC values in the 60°/s testing condition with the largest absolute peak torque generation occurring in the less involved limb. Since the exercise target torques used in the eccentric PRE were based on the initial MVC of each limb, the higher exercise workload of the less involved limb may partially explain the increased hypertrophic response of the left rectus femoris. However, both lower extremities exhibited substantial strength increases, with the more involved limb experiencing the largest relative increase in MVC levels across testing conditions despite showing minimal hypertrophic adaptations. This observation may reflect the concomitant neurological adaptations to the eccentric exercise stimulus (59).

The specificity of the eccentric PRE regimen may confer an advantage to people with knee OA since difficulty in descending a curb or a flight of stairs is a common patient complaint (60). Eccentric exercise may aid the performance of this mobility task since stair descent requires adequate eccentric knee extensor control and joint excursion that is unrestricted by pain. The Neurocom^®^ records the time during single limb support during the Step Up and Over test and the ipsilateral knee force produced during the maneuver. However, movement time during ambulation and step ascent/descent may be confounded in a variable manner by the impairments associated with OA. For example, patients that exhibit an antalgic gait pattern may have a rapid stair descent time in an effort to minimize the duration of unilateral weight bearing on the more affected side. By contrast, when muscle weakness is a prominent impairment in people with OA, then step ascent time may be prolonged secondary to low strength levels that are very close to the threshold level of force needed to accomplish the task. Moreover, the self-selected movement time during the Step Up and Over task may vary based on the selected movement strategy in higher functioning individuals. Those with ample strength reserves relative to the task may opt to complete the task as rapidly as possible, or they may choose to employ a deliberate and controlled step descent to minimize impact upon heel strike. The patient featured in this report exhibited similar mean pre and post-exercise movement times, with the longest single trial movement time duration occurring with the more involved limb during Week 12. The recorded slower post-exercise movement time may be the result of greater eccentric knee control upon step descent or simply a variation in movement strategy. These clinical observations should not detract from the demonstrated value of the Step Up and Over test in other patient populations. Investigators have shown that the test can discriminate between control subjects and those with ACL-deficient knees (37) or Parkinson disease (38), respectively. Nevertheless, our clinical experience suggests that the interpretation of the standard scores obtained from the Step Up and Over test may require more study in people with knee OA. It is important to note that the COG displacement trajectories afforded by the Neurocom^®^ force plate system provided useful information. This force plate system has a relatively high degree of clinical utility given its user-interface and unique dimensions (two adjacent 150 × 23-cm force plates), and the COG displacement associated with stepping appears to be a more appropriate assessment variable given the presumed effect of OA symptoms on single limb weight bearing capacity and step length.

**Figure 7:**
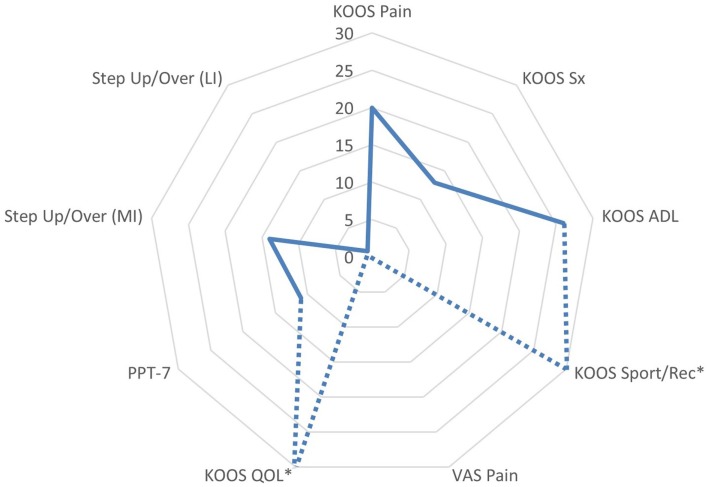
**Changes in functional assessments and pain scores following the submaximal eccentric progressive resistance exercise regimen**. The radar graph features the delta percentage values of the Knee Injury and Osteoarthritis Outcome Score (KOOS) Subscale Scores, the center of gravity linear displacement trajectories derived from the Step Up and Over test, and the visual analog scale pain rating. The largest post-intervention responses were observed in KOOS QOL and Sport/Rec subscales (*note: the change in these values exceeded 100%, and are estimated on the graph in order to maintain the graph scale and allow for comparisons among the remaining outcomes). Additionally, the VAS Pain rating was stable throughout the 12-week intervention period, whereas the KOOS Pain subscale improved by 20% (ADL, activities of daily living; QOL, quality of life; sport/rec, sports and recreation; Sx, symptom; PPT-7, Physical Performance Test; MI, more involved limb; LI, less involved limb; VAS, visual analog scale).

The patient appeared to have a favorable response to eccentric PRE based on his self-assessment of OA disease status and HRQL (Figure 7). All of the KOOS subscale post-exercise scores were increased with 4 out of 5 subscales meeting patient improvement criteria based on either the MPCI or MDC (Table 1). The patient recorded the largest improvements in the KOOS Sport/Rec and QOL subscales, which is consistent with the published literature describing these subscales as the most as responsive sections of the KOOS (effect size, 0.98 and 1.32, respectively) (45, 61). One methodological challenge associated with the use of the KOOS is the expression of OA disease status as five subscales rather than a total score. Given that construct validity may be stronger in the QOL subscale in comparison with the Sport/Rec subscale (45, 61), the QOL subscale may merit consideration as single primary outcome in observational studies and clinical trials. Another item of interest concerning the patient’s self-reported status was his rating of pain using a standard numeric VAS and the KOOS Pain subscale. Gür and colleagues (21) reported that patients with knee OA who performed combined eccentric-concentric isokinetic exercise had better functional outcomes in comparison to patients engaging in concentric-only isokinetic exercise. However, the concentric-only isokinetic exercise group exhibited better post-exercise pain relief in comparison to the patients that participated in the combined eccentric-concentric isokinetic exercise regimen. In this study, the pain rating using the VAS was stable throughout the exercise period (6 out of 10), while the Pain subscale score improved from 42 to 50. The improvement in Pain subscale was meaningful only based on the criterion related to the estimated MPCI, rather than the minimal detectable difference derived from a sample of people with knee OA (46). Nevertheless, this clinical observation raises the point that questionnaires that capture multidimensional aspects of pain may have conceptual and clinimetric advantages over a VAS rating of pain. While the post-exercise pain relief in people with knee OA dissipates with detraining, further investigation that characterizes the influence of exercise mode and intensity on patient pain ratings following exercise may help to optimize PRE program design (7, 11, 13).

## Concluding Remarks

The patient featured in this report appeared to have positive physiological adaptations to eccentric strength training without an exacerbation of his knee OA symptoms. His apparent improvement in knee extensor muscle size, tissue quality, and strength may have influenced his improved post-exercise status concerning functional performance and self-reported disease status and HRQL. All case reports constitute a detailed account of clinical practice, and cannot be used to establish causal relationships between interventions and patient outcomes. In addition, patients with significant comorbid factors and advanced age may have a differential response to the eccentric PRE stimulus described in this report. Nevertheless, this report served as a proof-as-concept endeavor that demonstrated the safe application of the eccentric PRE in a veteran with symptomatic and radiographic evidence of bilateral knee OA.

Continued investigation of the efficacy of eccentric PRE for older adults with knee OA may be merited. Additional pilot studies and observational trials will be needed to better understand the patient response to eccentric PRE based on estimates of OA severity and levels of patient disability. Further inquiry concerning exercise adherence in supervised and non-supervised settings would also aid the adoption of formalized PRE regimens in the management of knee OA. In general, traditional isoinertial strengthening approaches (i.e., exercise with an emphasis on concentric muscle actions) are more readily adaptable for home exercise programs in comparison to the eccentric training paradigm. Consequently, developmental work may be needed to refine eccentric PRE regimens for home use by patients with a wide range of physical abilities.

Regarding the outcome measures featured in this report, the use of diagnostic musculoskeletal ultrasound provided important information about post-exercise changes in muscle tissue morphology and had a low patient and practitioner burden. Moreover, the novel approach used in this report to objectively assess step ascent/descent performance using the Neurocom^®^ Step Up and Over test appeared to reveal differences between the more involved and less involved limb. However, more study will be needed to understand the clinical and research utility of this assessment method for people with knee OA. Finally, the KOOS questionnaire was a valuable tool in the clinical management of the patient. The QOL subscale may prove to be useful as a primary outcome measure in this patient population, and the multidimensional Pain scale may offer some advantages over the use of simple VAS ratings of pain related to knee OA.

## Conflict of Interest Statement

The authors declare that the research was conducted in the absence of any commercial or financial relationships that could be construed as a potential conflict of interest.
